# A225 ASSESSMENT OF BEDSIDE INTESTINAL ULTRASOUND IN MONITORING CROHN’S DISEASE ACTIVITY

**DOI:** 10.1093/jcag/gwad061.225

**Published:** 2024-02-14

**Authors:** N Ashrafinia, T Guzowski, S Fowler

**Affiliations:** University of Saskatchewan, Saskatoon, SK, Canada; University of Saskatchewan, Saskatoon, SK, Canada; University of Saskatchewan, Saskatoon, SK, Canada

## Abstract

**Background:**

Bedside intestinal ultrasound (IUS) is an evolving modality in monitoring disease activity and assessing complications in inflammatory bowel disease (IBD). The easy-tolerability and immediate accessibility to results for expedited clinical decision-making contribute to the growing global attraction of IUS integration into IBD care. Fecal calprotectin (FC) is a known, non-invasive tool to assess disease activity in IBD. However, it comes with limitations.

**Aims:**

To assess the efficacy of IUS in monitoring Crohn’s Disease (CD) compared to FC level.

**Methods:**

We conducted a retrospective observational study in patients with confirmed CD at a certified IUS gastroenterologist’s outpatient clinic in Saskatoon. Patients’ charts undergoing IUS between February 2022 and April 2023 were reviewed. Patients with CD and available FC results within a month of IUS performance were identified. Age, gender, disease character, clinical symptoms (Harvey-Bradshaw Index (HBI)), FC levels, and IUS bowel wall thickening (BWT) were collected. The cut-off for FC levels and BWT for disease activity were ampersand:003E250 μg/g and ampersand:003E3 mm, respectively. The correlation between IUS BWT and FC levels was analyzed based on the Pearson correlation.

**Results:**

Among a total of 178 chart reviews, 71 patients were identified. The median age was 49 years, 52.1% were males and 47.8% were females. 35 (49.3%) ileal CD, 35 (49.3%) ileocolonic and 1 (1.4%) colonic CD were identified. The HBI score verified 67.6% mild disease, 19.7% moderate, 11.2% in remission, and 1.4% severe disease. The Pearson correlation coefficient (r) between FC levels and BWT findings on IUS was r = 0.29 (P ampersand:003C0.0001), indicating a weak positive correlation.

**Conclusions:**

A positive but weak correlation exists between bowel wall thickening in IUS and FC level in monitoring CD. Recognizing FC limitations, future multi-center, larger cohort studies need to be done to investigate this correlation further.

**Funding Agencies:**

None

